# Preparation of novel karaya gum derived covalent immobilizers via polyethylene-imine and glutaraldehyde processing

**DOI:** 10.1038/s41598-026-45030-1

**Published:** 2026-04-11

**Authors:** Marwa I. Wahba

**Affiliations:** 1 Department of Chemistry of Natural and Microbial Products, National Research Centre, El-Behooth St., Dokki, Giza, Egypt; 2 Centre of Scientific Excellence-Group of Advanced Materials and Nanotechnology, National Research Centre, El-Behooth St., Dokki, Giza, Egypt

**Keywords:** Karaya gum, Polyethylene-imine, Glutaraldehyde, β-galactosidase, Immobilizers, Biochemistry, Biotechnology, Chemistry, Materials science

## Abstract

In this study, novel karaya gum (KG) based covalent immobilizers were developed. Initially, KG was mixed with agar so as to acquire a handle-able hydrogel, which was subsequently processed with polyethylene-imine (PE) and glutaraldehyde (GA). Optimization via Box-Behnken Design (BBD) revealed that the optimal PE/GA processing should be accomplished using a 6.1% PE solution of pH 8.5 and a 5.8% GA solution. The PE/GA processing was monitored via FTIR and SEM. The optimized GA/PE/KG-agar matrix immobilized β-galactosidase (β-GL) with immobilization efficiencies reaching up to 68.73%. The temperature and pH profiles of the GA/PE/KG-agar immobilized β-GL (iβ-GL) were compared with those of the free β-GL. The iβ-GL exhibited improved thermal stability, evidenced by its more escalated t_1/2,_ D-values, and ΔG values. The iβ-GL also exhibited finer solvent and heavy-metals stabilities than did its free homologue. Furthermore, the iβ-GL provided 95.11% activity during its 23rd reusability cycle and 92.86% activity after 9 weeks storage period. Finally, the GA/PE/KG-agar iβ-GL degraded whey permeate lactose for 6 successive 24 h cycles.

## Introduction

Karaya gum (KG) is procured from the exudate of *Sterculia urens* trees^1^. KG is safe and biodegradable. Furthermore, it is characterized by its availability, low cost, escalated water-uptake and its fine thickening, and emulsifying qualities^1–3^. Accordingly, it is extensively utilized as food adjuvent and pharmaceutical ingredient^2,3^. KG has also been inspected for the formulation of wound dressings^4^, gastric-retention systems, trans-dermal patches^5^, and tissue-engineering scaffolds^6^. KG derived packaging-materials were also inspected^7^.

KG comprises a backbone of α-D-galacturonic acid and α–L-rhamnose. The backbone α-D-galacturonic acid entities could be attached to side chains of either β-D-glucuronic acid or β-D-galactose. The backbone α–L-rhamnose entities could also be attached to β-D-galactose side chains. Furthermore, KG is partially acetylated with ≈8% acetyl residues as compared to 55–60% neutral sugars and 37–40% acid sugars^5,7^. These acid sugars would provide KG with anionic qualities; and accordingly, KG could interact with and bind to cationic entities, such as polyethylene-imine (PE). Noteworthy, interaction with PE was the initial step of converting altered anionic polysaccharides, such as calcium pectinate, chia gum, and gellan gum into covalent immobilizers. The interactions amongst the polysaccharides anionic entities and PE cationic amine entities, led to the binding of PE to the polysaccharide matrix. The bound PE provided nucleophilic amino entities for the binding of glutaraldehyde (GA). GA was then utilized to covalently conjugate enzymes^8,9^.

Immobilization is a protocol whose original goal was to simplify enzymes recovery and permit their reuse^10^ as it entails conjugating enzymes to insoluble matrices that can be easily withdrawn from reactions. Nonetheless, other beneficial immobilization impacts were reported, such as contributing to enzymes purification, promoting enzymes activity & stability, tuning enzymes selectivity, and also impeding their inhibition^10^. Immobilization could be accomplished through altered techniques, such as adsorption, entrapment, and also covalent conjugation. However, during covalent conjugation, enzymes are coupled via the stable, irreversible covalent links^10^. As a result, the leakage of the conjugated enzymes would be hindered, and the fabricated bio-catalysts would retain their functionality for long. Furthermore, the multi-point covalent links created amidst the immobilizers and their conjugated enzymes would rigidify the enzymes configuration, making them more resistive against distorting factors^11^, such as raised temperatures, and this would contribute to the retention of the bio-cataysts functionality. Consequently, the development of covalent immobilizers based on KG would be highly beneficial, particularly given that such systems have not yet been documented in literature.

However, the fabrication of KG-based covalent immobilizers necessitates the prior formulation of KG into a solid hydrogel matrix. In one reported approach, a KG-derived hydrogel was synthesized through free radical polymerization by combining KG with two monomers (acrylamide and N-isopropylacrylamide) alongside a crosslinking agent (2-bis-methacryloyloxy-ethyl-phosphate) and an initiator (potassium persulfate)^3^. Alternatively, KG was blended with a carboxymethylated derivative of locust bean gum (CM-LBG), specifically modified to facilitate coordination interactions with Al^3^⁺ ions. This KG-(CM-LBG) blend was subsequently transformed into hydrogel beads through its dropwise addition into an Al^3^⁺ solution^5^. However, Al^3^⁺ poses neurotoxic risks upon biological absorption^12^, necessitating the exploration of alternative strategies for KG hydrogel formation.

In this study, KG based hydrogel was developed by incorporating agar, which undergoes a structural transition from coil-to-helix upon cooling, thereby enabling gelation without chemical cross-linkers. Agar is a widely used food additive^13^ and is also recognized for its high mechanical strength^14^, a property that would facilitate the development of handle-able KG-agar hydrogels. Importantly, agar exhibits anionic characteristics owing to its agaropectin content^13^. Thus, it might interact with the cationic PE during the PE/GA activation of KG-agar. In order to reduce agar involvement in the PE/GA activation process and enable a more thorough examination of KG effectiveness as an immobilizer, agar was utilized at 1% concentration whereas a 3% KG was adopted.

The GA/PE/KG-agar immobilizers, introduced in this study, would constitute hetero-functional immobilizers. PE would ionically adsorb the enzymes^15^ whereas GA would mediate their covalent conjugation^16^. Nonetheless, ionic adsorption occurs significantly faster than covalent conjugation^10^. Accordingly, the initial interactions between the acquired immobilizers and the enzymes would, mostly, be ionic, whereas the covalent links would be established later^16^. Such speedy inceptive ionic adsorption would quickly remove the enzymes from solution, and this would help retain the enzymes activity^16^.

The developed GA/PE/KG-agar matrix was inspected as β-D-galactosidase (β-GL) immobilizer. β-GL, being a lactose degrading enzyme, is essential in the fabrication of low-lactose and no-lactose dairy products for the consumption of the lactose-intolerant^17^. The β-GL catalyzed lactose degradation could also serve to valorize whey and encourage its industrial utilization^9^. Furthermore, β-GL can present transglycosylase activity which can be utilized to fabricate galacto-oligosaccharides (GOS)^17,18^. GOS, being prebiotics, could be merged within foods to encourage the growth of the advantageous gut microbiota^9^.

In this work, KG, a readily available and cost-effective polysaccharide, was successfully transformed into a covalent immobilizer for the first time. This transformation was achieved by initially fabricating a handle-able KG-based hydrogel via integration with agar, followed by sequential processing with PE and GA. The obtained GA/PE/KG-agar matrix was used to immobilize β-GL. The PE/GA processing was adjusted via BBD in order to maximize the immobilized β-GL (iβ-GL) activity. Moreover, the impact of immobilization on iβ-GL was assessed by examining the temperature and pH profiles, as well as, the thermal, solvent, and heavy metals stabilities of the GA/PE/KG-agar iβ-GL and its free homologue. The iβ-GL storage stability and reusability were also studied. Finally, the GA/PE/KG-agar iβ-GL was applied in whey permeate lactose degradation.

## Materials and methods

### Materials

KG, PE (Mw≈800), *Aspergillus oryzae* β-GL, and GA (50%, 5.6 M solution) were obtained from Sigma-Aldrich, Germany. All other fine chemicals were of Analar grade or equivalent quality.

### Methods

#### The GA/PE/KG-agar immobilizers preparation

A 4% (w/w) KG and 4% (w/w) agar solutions were individually prepared in distilled water via stirring their powders at room-temperature, and in a boiling water-bath, respectively. Following complete dissolution of KG, its solution was placed in a 50 °C water-bath for 10 min, and then it was mixed with the agar solution at a 3 KG:1 agar ratio so that the final concentrations were 3% (w/w) KG and 1% (w/w) agar. The KG-agar mixture (≈12 gm) was poured into a 9 cm plastic petri-dish, and was left to cool and solidify. Afterwards, the KG-agar hydrogel was removed from the petri-dish and was cut into discs (≈5.47 mm in diameter) with a cork-borer. The KG-agar discs were then soaked within PE solution for 2 h, meticulously laved, and soaked within GA solution for 1 h. Finally, the GA/PE/KG-agar discs were meticulously laved, and were stored refrigerated in distilled water till β-GL conjugation. Notably, a Box-Behnken Design (BBD) was implemented where the PE concentration, the PE pH and the GA concentration were the independents variables and the iβ-GL activity was the dependent variable (Table 1). The BBD aimed to disclose the optimal GA/PE/KG-agar immobilizers preparation conditions, which would provide the uttermost iβ-GL activity.

**Table 1:** Independent variables involved in the BBD, their units and levels, and the dependent variable with its unit.

Variable type	Name	Unit	Low level (− 1)	Central level (0)	High level (+ 1)
Independent variable	PE pH		7.0	7.8	8.6
	PE concentration	% (w/w)	0.5	3.5	6.5
	GA concentration	% (v/v)	1.0	3.5	6.0
Dependent variable	iβ-GL activity	Ug^−1^	N/A*	N/A	N/A

* Not applicable.

#### β-GL immobilization

Powdered *A. oryzae* β-GL was dissolved in 0.1 M citrate–phosphate buffer (pH 4.8). This β-GL solution was mixed with the GA/PE/KG-agar discs (10:1, v/w ratio), and the mixture was kept at room-temperature overnight. Afterwards, the discs were meticulously laved with distilled water to discard the un-conjugated β-GL, and their activity was inspected.

#### β-GL activity inspection

A 0.1 M citrate–phosphate buffer (pH 4.8) served as the assay medium for dissolving free β-GL or suspending β-GL-loaded GA/PE/KG-agar discs, and was additionally employed in the preparation of a 200 mM lactose solution. The assay was performed via mixing 0.5 ml assay buffer containing either the free β-GL or 2 loaded GA/PE/KG-agar discs with 3.5 ml lactose solution. The assay proceeded for 15 min in a 37 °C agitating water-bath. Afterwards, some of the reaction solution was removed and heated for ≈10 min in a boiling-water-bath. The glucose was then estimated in this reaction solution via commercial glucose kits. The detection of 1 μmol glucose/min under the reaction conditions described above corresponded to one β-GL unit (U). The iβ-GL activity (Ug^−1^) was presented in relation to the weight of the GA/PE/KG-agar discs, which was recorded before β-GL loading as this was the weight which determined the volume of loaded β-GL solution.

#### Monitoring the PE/GA processing of the KG-agar discs

##### Scan electron microscope (SEM)

The lyophilized KG-agar discs, PE/KG-agar discs, and GA/PE/KG-agar discs were visualized via SEM, without any further processing.

##### Fourier transform infrared (FTIR)

The KG-agar discs, the PE/KG-agar discs, and the GA/PE/KG-agar discs were individually grind using a mortar pestle. They were then dried (≈60 °C) and further grind to reach the fine powder consistency. The powders FTIR spectra were inspected amongst 4000–400 cm^−1^ range while adopting the KBr pellet technique.

#### Chemical stability of the GA/PE/KG-agar immobilizers

In this experiment, the release of GA from the GA/PE/KG-agar immobilizers was monitored to ensure their chemical stability. Around 0.85 gm of the GA/PE/KG-agar discs was soaked in 5 ml 0.1 M citrate–phosphate buffer (pH 4.8). This mixture was incubated in a rotating incubator (37 °C, 100 rpm) for 19 h. The soaking citrate–phosphate buffer was then tested using DNPH (2,4-dinitrophenylhydrazine) to detect any released GA. The freshly prepared DNPH solution^19^ (10 ml) was mixed with 1 ml of the soaking citrate–phosphate buffer. This mixture was incubated for 1 h in a 30 °C water-bath, and then it was centrifuged. The O.D. of the supernatant, which contained the un-reacted DNPH, was recorded at 360 nm^20^ in comparison with a blank that comprised 1 ml of water instead of the soaking buffer. A standard curve was constructed with varying concentration of the DNPH solution.

#### pH profiles

The β-GL activity inspection was undergone. However, altered buffers were adopted, such as the 0.1 M KCl–HCl buffer (pH 2.4), and the 0.1 M 0.1 M citrate–phosphate buffers (pHs 3.4–7.7). The most escalated activity was denoted as 100%, and all other activities were given relative to it.

#### Temperature profiles

The β-GL activity inspection was undergone. However, the temperature was varied (37–70 °C). The most escalated activity was denoted as 100%, and all other activities were given relative to it.

#### Thermal stability

The 0.5 ml assay buffer aliquots, which contained either the free β-GL or 2 loaded GA/PE/KG-agar discs, were placed in test-tubes within heated water-baths (58, 60, 62, & 64 °C). At precise timings, the test-tubes were removed, air-cooled for ≈ 30 s, and placed in a 37 °C water-bath. Lactose was immediately added and the activity inspection was undergone. The obtained activities (residual activities) were denoted in relation to the inceptive activities, that were recorded for non-thermally incubated specimens. Thereafter, a plot of log (residual activity percents) vs thermal incubation duration (min) was created, and its -slope was regarded as k_d_ (first order thermal denaturation rate constant). The k_d_ values were then utilized to reckon the half-lives (t_1/2_) and decimal reduction times (D-values) as follows^9^:

1 $${\mathrm{t}}_{{{1}/{2}}} = {\mathrm{ln2}}/{\mathrm{k}}_{{\mathrm{d}}}$$

2 $${\mathrm{D-value}} = \ln 10/{\mathrm{k}}_{{\mathrm{d}}}$$

The log (D-values) were plotted versus temperature (°C), and the -1/slope in this plot corresponded to the z-value. On the other hand, the (-slope/R) of the plot of ln (k_d_) versus 1/temperature (K) corresponded to the activation energy of β-GL thermal denaturation (E_d_). The variations in enthalpy (ΔH), entropy (ΔS), and Gibb’s free energy (ΔG) betwixt the active and the thermal denatured β-GLs were reckoned^9^:

3 $$\Delta {\mathrm{H}} = {\mathrm{E}}_{{\mathrm{d}}} {-}{\text{ RT}}$$

4 $$\Delta {\mathrm{G}} = - {\mathrm{RTln}}\left( {{\mathrm{k}}_{{\mathrm{d}}} *{\mathrm{h}}/{\mathrm{k}}_{{\mathrm{B}}} *{\mathrm{T}}} \right)$$

5 $$\Delta {\mathrm{S}} = \left( {\Delta {\mathrm{H}} - \Delta {\mathrm{G}}} \right)/{\mathrm{T}}$$

T was the temperature in K, R was the universal gas constant (8.314Jmol^−1^ K^−1^), h was the Planck constant (11.04 * 10^–36^ Jmin), and k_B_ was the Boltzman constant (1.38*10^−23^JK^−1^).

#### Influence of heavy-metals on the stability of the free and the GA/PE/KG-agar iβ-GLs

HgCl_2_, Cr_2_(SO_4_)_3_, and Al(NO_3_)_3_ solutions as well as PbSO_4_ and FeCl_3_ suspensions were prepared in 0.1 M citrate–phosphate buffer (pH 4.8) at 20 mM concentrations. Afterwards, 0.25 ml volumes of these solutions were individually added to 0.25 ml 0.1 M citrate–phosphate buffer (pH 4.8), which contained either the free β-GL or 2 loaded GA/PE/KG-agar discs. These mixtures were placed for 2 h at room temperature prior to their activity inspection. The recorded activities were denoted relative to the activity of the control, which was mixed with 0.25 ml buffer rather than the heavy-metals solutions.

#### Solvent stability of the free and the GA/PE/KG-agar iβ-GLs

Altered solvents (0.25 ml) were individually mixed with 0.25 ml 0.1 M citrate–phosphate buffer (pH 4.8), which contained either the free β-GL or 2 loaded GA/PE/KG-agar discs. These mixtures were placed for 2 h at room temperature prior to their activity inspection. The recorded activities were denoted relative to the activity of the control, which was mixed with 0.25 ml buffer rather than the solvent.

#### GA/PE/KG-agar iβ-GL storage stability

Around 2 gm of the GA/PE/KG-agar discs were loaded with β-GL. The activity of some of these loaded discs was inspected and denoted as 100%. The rest of the loaded discs were refrigerated whilst being immersed in 0.1 M citrate–phosphate buffer (pH 4.8). After specific periods, the activity of some of these loaded discs was inspected, and presented in relation to the inceptive 100% activity.

#### GA/PE/KG-agar iβ-GL reusability

The activity of some of the β-GL loaded discs GA/PE/KG-agar was inspected and denoted as 100%. These discs were then laved twice with the assay buffer, and their activity was re-inspected. The activities were presented in relation to the inceptive 100% activity. Notably, this study was conducted over four consecutive days, with the discs refrigerated in between. On the first day, 11 runs were performed whereas 4 runs were conducted on each of the following 3 days.

#### Degrading whey permeate lactose via the free and the GA/PE/KG-agar iβ-GLs

Whey permeate (WP) was obtained from a local cheese manufacturing facility and subjected to thermal treatment by immersion in a water-bath maintained at 80 °C for a duration of 2 h. Following heat processing, the sample was stored under frozen conditions until required. Prior to experimental use, the WP was thawed and subsequently decanted to eliminate any precipitated proteinaceous material. The pH of the WP was then modified to 4.6, and it was filtered. A volume of 4 ml of the WP was added to β-GL loaded GA/PE/KG-agar discs (1.05 U), and the mixture was incubated in a rotating incubator (45 °C, 100 rpm) for 24 h. The treated WP was then collected for glucose quantification, while the β-GL loaded GA/PE/KG-agar discs were laved twice with distilled water. Afterwards, fresh WP was added to the discs to initiate a subsequent cycle of lactose degradation. For comparative purposes, free β-GL was also employed in parallel experiments: 200 µl of the free β-GL, which provided 1.13 U activity, was added to 4 ml of WP and incubated under identical conditions (45 °C, 100 rpm, 24 h). After incubation, the reaction was terminated by immersing the sample in a boiling water bath for approximately 10 min, followed by glucose measurement, with corrections applied to account for dilution due to the added volume of the free enzyme. The inceptive WP lactose was also quantified^21^ in order to reckon the lactose degradation proficiency (LDP) as follows:

6 $${\mathrm{The}}\;{\mathrm{released}}\;{\mathrm{glucose}}\left( {\upmu {\mathrm{mol}}/{\mathrm{ml}}} \right)/{\mathrm{inceptive}}\;{\mathrm{WP}}\;{\mathrm{lactose}} \left( {\upmu {\mathrm{mol}}/{\mathrm{ml}}} \right)*{1}00$$

### Statistical analysis

The BBD was inspected via Design Expert 13. Moreover, statistically significant differences (*p*-value < 0.05) amidst altered sets of results were inspected via combining one way analysis of variance (ANOVA) with Tuckey-Kramer post-hoc. Notably, all experiments were designed as triplicates, and the standard error was computed as the standard deviation divided by the square root of the number of specimens.

## Results and discussion

### The BBD

**Fig. 1:**
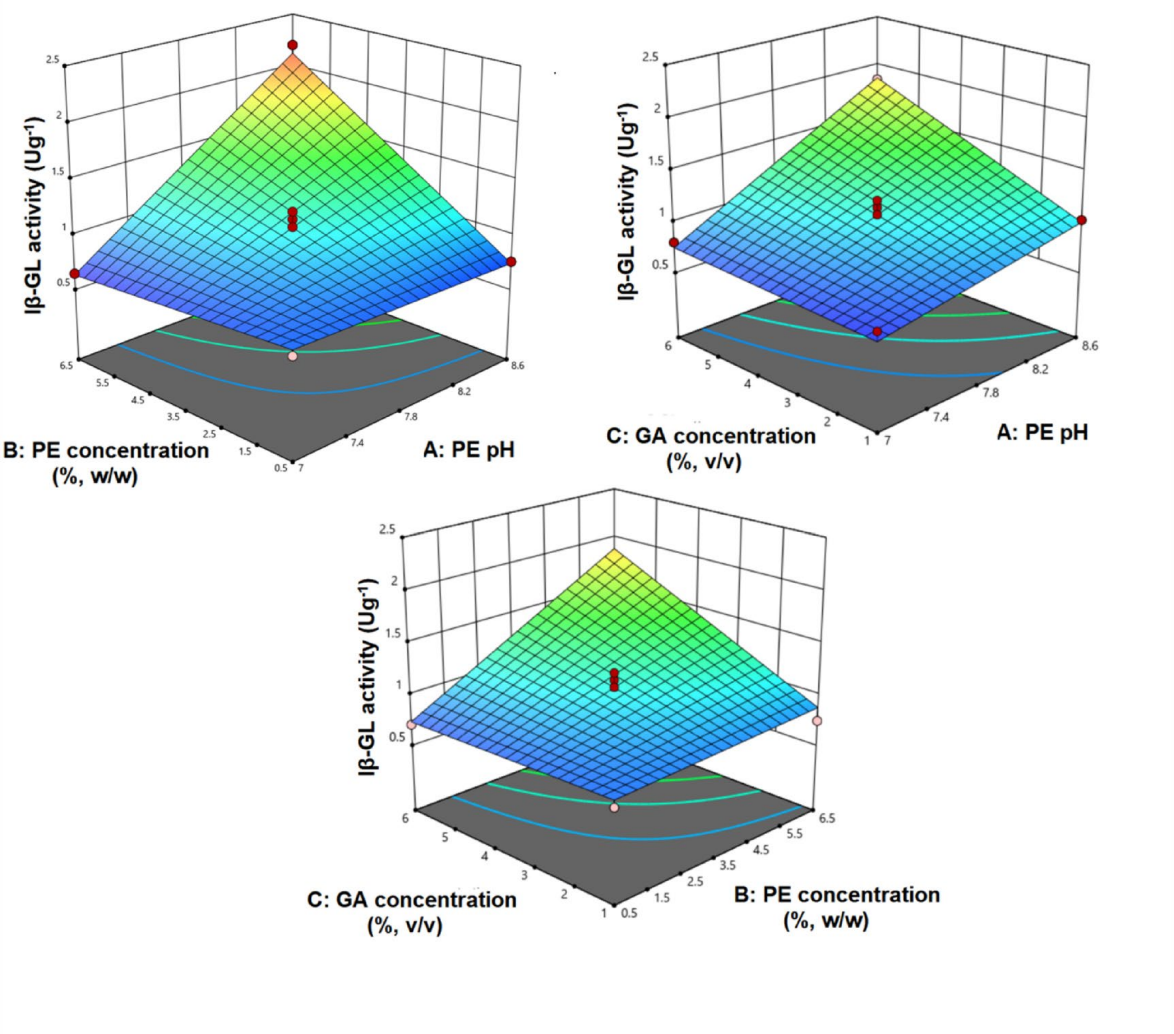
3D graphs inspecting the iβ-GL activities that would be obtained at altered PE pH (**A**), PE concentration (**B**) and GA concentration (**C**). In each figure, two of the aforementioned factors were altered whereas the third was fixed (0 level).

**Fig. 2:**
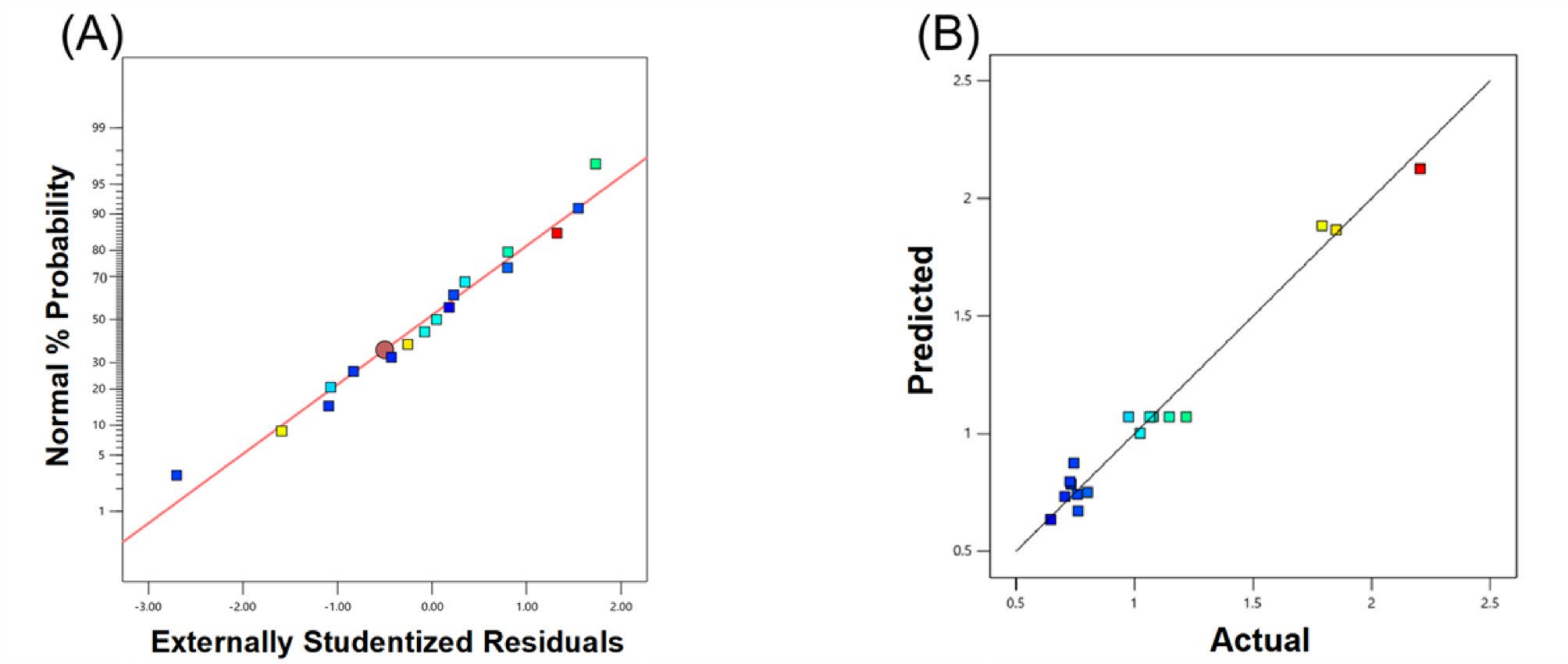
(**A**) Normal plot of residuals and (**B**) Predicted versus actual plot.

The BBD (Table 2) was analyzed via a two-factors-interactions model. The model equation was given as Eq. (7), and its 3D graphs were presented in Fig. 1. The model *p*-value and R^2^ amounted to < 0.0001 and 0.9736, respectively, and this reflected the model significance and its capability to interpret the BBD data. Furthermore, the normal plot of residuals (Fig. 2A) disclosed that the residuals were reasonably distributed along the theoretical normal straight line, which reflected the normal distribution of the model residuals. The predicted versus actual plot (Fig. 2B) also disclosed that the obtained iβ-GL activities were in good agreement with the corresponding model-predicted values as most points were closely distributed around the 45° reference line, and this further confirmed the adequacy of the selected model.

7 $${\mathrm{i}}\upbeta {\mathrm{-GL}} = 1.0700 + 0.3616{\mathrm{A}} + 0.3075{\mathrm{B}} + 0.2363{\mathrm{C}} + 0.3831{\mathrm{AB}} + 0.1964{\mathrm{AC}} + 0.2680{\mathrm{BC}}$$

**Table 2:** Settings of the BBD experimental runs, and the iβ-GL activities obtained at each run.

Run	A: PE pH	B: PE concentration (%, w/w)	C: GA concentration (%, v/v)	iβ-GL activity (Ug^−1^)
1	8.6 (+ 1)	3.5 (0)	1 (− 1)	1.02
2	7.8 (0)	0.5 (− 1)	1 (− 1)	0.73
3	8.6 (+ 1)	0.5 (− 1)	3.5 (0)	0.76
4	7.8 (0)	3.5 (0)	3.5 (0)	1.08
5	8.6 (+ 1)	6.5(+ 1)	3.5 (0)	2.20
6	7.8 (0)	3.5 (0)	3.5 (0)	1.22
7	7 (− 1)	6.5(+ 1)	3.5 (0)	0.65
8	7.8 (0)	3.5 (0)	3.5 (0)	0.97
9	7.8 (0)	6.5(+ 1)	6 (+ 1)	1.79
10	7 (− 1)	3.5 (0)	6 (+ 1)	0.80
11	8.6 (+ 1)	3.5 (0)	6 (+ 1)	1.85
12	7.8 (0)	0.5 (− 1)	6 (+ 1)	0.71
13	7.8 (0)	3.5 (0)	3.5 (0)	1.06
14	7 (− 1)	0.5 (− 1)	3.5 (0)	0.73
15	7.8 (0)	6.5(+ 1)	1 (− 1)	0.74
16	7.8 (0)	3.5 (0)	3.5 (0)	1.15
17	7 (− 1)	3.5 (0)	1 (− 1)	0.76

**Fig. 3:**
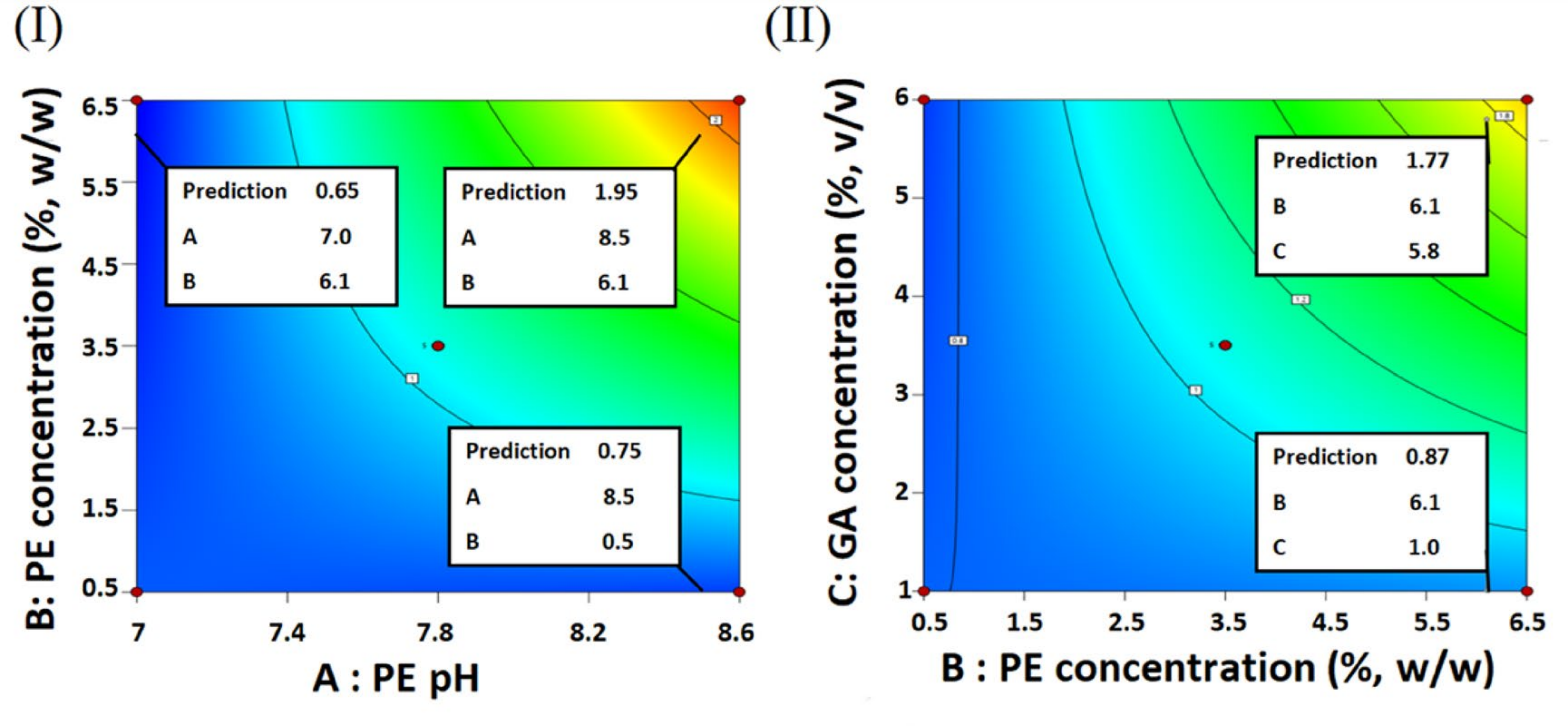
Contour plots inspecting the iβ-GL activities that would be obtained at altered PE pH (**A**), PE concentration (**B**) and GA concentration (**C**). In each figure, two of the aforementioned factors were altered whereas the third was fixed (0 level).

Table 3 revealed that all the factors and their interaction terms were significant. The significant impact of PE pH and PE concentration could be verified from Fig. 3I which inspected the impacts of varying PE pH (A) and PE concentration (B) at a fixed GA concentration (C) (0 level; 3.5%). Varying the PE pH from 7 to 8.5 would trigger a 3.0 fold escalation in the iβ-GL activity, if 6.1% PE concentration was adopted whereas varying the PE concentration from 0.5% to 6.1% would trigger a 2.6 fold escalation in the iβ-GL activity, if 8.5 PE pH was adopted. As regards to the impact of GA concentration, Fig. 3II revealed that varying the GA concentration from 1% to 5.8% would trigger a 2.0 fold escalation in the iβ-GL activity, if 6.1% PE concentration and 7.8 PE pH (0 level) were adopted.

**Table 3:** Analysis of variance (ANOVA) for the model which was adopted to analyze the BBD.

Source	SS^a^	DF^b^	MS^c^	F-value	*P*-value
Model	3.2800	6	0.5462	61.42	< 0.0001
A-PE pH	1.0500	1	1.05	117.58	< 0.0001
B-PE conc	0.7562	1	0.7562	85.03	< 0.0001
C-GA conc	0.4468	1	0.4468	50.24	< 0.0001
AB	0.5869	1	0.5869	65.99	< 0.0001
AC	0.1542	1	0.1542	17.34	0.0019
BC	0.2873	1	0.2873	32.31	0.0002
Residual	0.0889	10	0.0089		
Lack of Fit	0.0557	6	0.0093	1.12	0.4796
Pure Error	0.0333	4	0.0083		
Cor Total	3.3700	16			

^a^Sum of squares.

^b^Degrees of freedom.

^C^Mean square.

The model analysis disclosed that 8.5 PE pH, 6.1% PE concentration and 5.8% GA concentration were optimal for the preparation of the GA/PE/KG-agar immobilizers. At these optimal settings, the model predicted that 2.52 Ug^−1^ iβ-GL activity would be obtained. Noteworthy, the design was loaded with 20.48 ± 0.26 Ug^−1^ β-GL and Table 4 disclosed that 2.09 Ug^−1^ and 3.06 Ug^−1^ iβ-GL activities were obtained by the optimal GA/PE/KG-agar immobilizers after being loaded with 18.46 Ug^−1^ and 23.36 Ug^−1^ β-GL, respectively. That is, the obtained values aligned with the model predicted value.

**Table 4:** Effect of varying the loaded β-GL activity on the recorded iβ-GL activity, the residual β-GL activity, the immobilization yield and the immobilization efficiency.

Loaded β-GL activity(A)	Recorded iβ-GL activity (B)	Residual β-GL activity (C)	Immobilization yield (%)	Immobilization efficiency (%)
Ug^−1^	Ug^−1^	Ug^−1^	((A-C)/A)*100)	((B/(A-C)*100)
5.99 ± 0.04	0.83 ± 0.05	4.78 ± 0.04	20.21	68.73
11.79 ± 0.14	1.45 ± 0.06	9.02 ± 0.18	23.49	52.49
18.46 ± 0.05	2.09 ± 0.07	13.29 ± 0.05	28.02	40.31
23.36 ± 0.28	3.06 ± 0.15	15.75 ± 0.22	32.57	40.24
29.79 ± 0.27	3.84 ± 0.18	19.12 ± 0.12	35.83	35.94

The substantial acid sugar content of KG (37–40%^7^) would enable the efficient ionic cross-linking with the abundant cationic PE moieties that would be presented by the optimal 6.1% PE solution. It should also be noted that proximate PE settings (pH 8.3 and concentration 6.15%) were optimal for the preparation of the GA/PE/gellan gum immobilizers. Moreover, the proximate 8.32 pH and 5.43% concentration were the optimal PE settings that were adopted during the preparation of the GA/PE/chia gum immobilizers^9^. Escalated PE concentrations were previously argued to induce the formulation of thick PE coatings around the supports owing to the rapid deposition of numerous PE moieties^15^. The thick PE coating, which would be formulated around the KG-agar discs upon utilizing the optimal escalated 6.1% concentration, would enhance the mechanical stability of the KG-agar films. Such enhancement would be essential given the deliberate reduction of agar, the primary gelling agent, to 1% to facilitate a more rigorous assessment of KG effectiveness as an immobilizer. In contrast, a 1.5% PE solution was optimal for processing the composite beads, which were formulated from 3% gellan-gum, 1.09% sodium-alginate, and 1.01% soy-protein-isolate. These composite beads comprised large contents of the gelling gellan-gum and sodium alginate^22^. Thus, they didn’t require the formulation of thick stabilizing PE coating during their PE/GA processing, and accordingly, a reduced PE concentration was sufficient to process them. Likewise, the beads, which comprised the gelling gellan-gum together with agar, were optimally processed with a 1.62% PE solution^23^.

Moreover, the thick PE coating would provide incremented reactive volume for interaction^15^ with the iβ-GL, and this could facilitate the formation of a substantial number of ionic interactions between the PE^15^ functionalized KG-agar discs and the iβ-GL. These ionic interactions would, mostly, be the initial binding events occurring between the GA/PE/KG-agar immobilizer and iβ-GL, as previously discussed. Consequently, the enhanced density of ionic linkages at the elevated PE concentration of 6.1% would promote β-GL conjugation to the GA/PE/KG-agar. Furthermore, the thick PE coating would provide larger amount of the nucleophilic amine entities, that are necessary for GA binding^16^. Thus, more GA moieties would bind to the 6.1% PE processed GA/PE/KG-agar immobilizers, enabling increased covalent attachment of β-GL moieties. Based on this analysis, it could be deduced that the structural composition of KG, particularly its high content of acidic sugars^7^, combined with the necessity for mechanical reinforcement via a thick PE coating around the KG-agar discs, led to the selection of the 6.1% PE solution as optimal for processing the GA/PE/KG-agar immobilizers. This optimal 6.1% PE would result in the formulation of a thick PE coating around the KG-agar films which would positively influence the amount of bound GA and also the amount of bound β-GL. Moreover, this thick PE coating would contribute to the improved iβ-GL stability in the presence of heavy-metal ions and organic solvents, a topic to be elaborated upon in subsequent sections.

Regarding the optimal 8.5 PE pH, Zhou et al.^24^ recorded an escalation in the absolute value of KG anionic zeta-potential as the pH was raised from 7 to 8, indicating that KG became more anionic after raising its pH beyond 7. This enhanced anionic character would strengthen the ionic interactions between KG and the cationic PE, providing a rationale for choosing pH 8.5 over pH 7 as the optimal PE pH. With respect to the optimal 5.8% GA concentration, proximate GA concentrations of 5.66% and 6.02% were recommended earlier during the preparation of the GA/PE/calcium pectinate and the GA/PE/chia gum immobilizers, respectively^9,25^. Moreover, 5% GA was adopted during the PE/GA processing of the carrageenan-calcium pectinate beads, and the composite beads, which were formulated from gellan-gum, sodium-alginate, and soy-protein-isolate^22,26^. These concentrations were higher than the 1% GA concentration, which was the least concentration stated by Monsan^27^ to conjugate > 5 g-atom carbon/g-atom nitrogen. Accordingly, at such GA (C_5_H_8_O_2_) concentrations, GA could bind to the PE functionalized immobilizers via the GA/GA/amino configuration, which is most reactive towards proteins amino moieties^16^, and this would promote the immobilizers reactivity towards the β-GL moieties.

### Monitoring the PE/GA processing of the KG-agar discs

#### SEM inspection

**Fig. 4:**
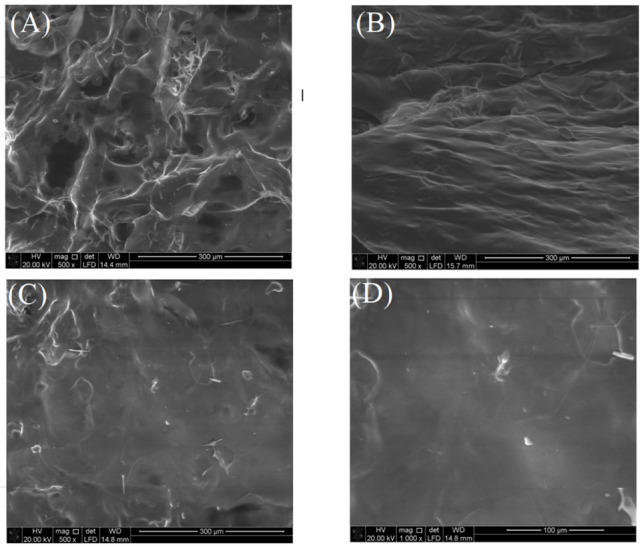
The surfaces of the lyophilized (**A**) KG-agar, (**B**) optimal PE/KG-agar, and (**C**) optimal GA/PE/KG-agar discs as appeared after a 500 X magnification via SEM. (**D**) The surface of the optimal GA/PE/KG-agar discs as appeared after a 1000 X magnification via SEM.

The lyophilized KG-agar discs surface exhibited pronounced irregularity, characterized by extensive wrinkling and porosity (Fig. 4A). Lyophilization is well-documented as a method for generating porous hydrogel structures^28^. Thus, the irregular porous morphology of the lyophilized KG-agar discs could be attributed to the lyophilization process employed for sample drying. A progressive reduction in surface topographical irregularities was evident following subsequent PE (Fig. 4B) and GA (Fig. 4C) processing. The thick PE coating, which was formulated around the KG-agar discs, might have reduced the deleterious effects of lyophilization. Moreover, GA cross-linking fortified the PE coating, and this led to a finer protection versus lyophilization deleterious effects. Consequently, the GA/PE/KG-agar discs displayed a pore-free surface with only slight corrugations, even under magnification of 1000X (Fig. 4D). This trend aligned with prior observations in which PE/GA processing similarly diminished surface roughness in lyophilized specimens, including gellan gum-alginate-soy protein isolate and calcium pectinate/whey-protein-isolate beads^8^.

#### FTIR inspection

**Fig. 5:**
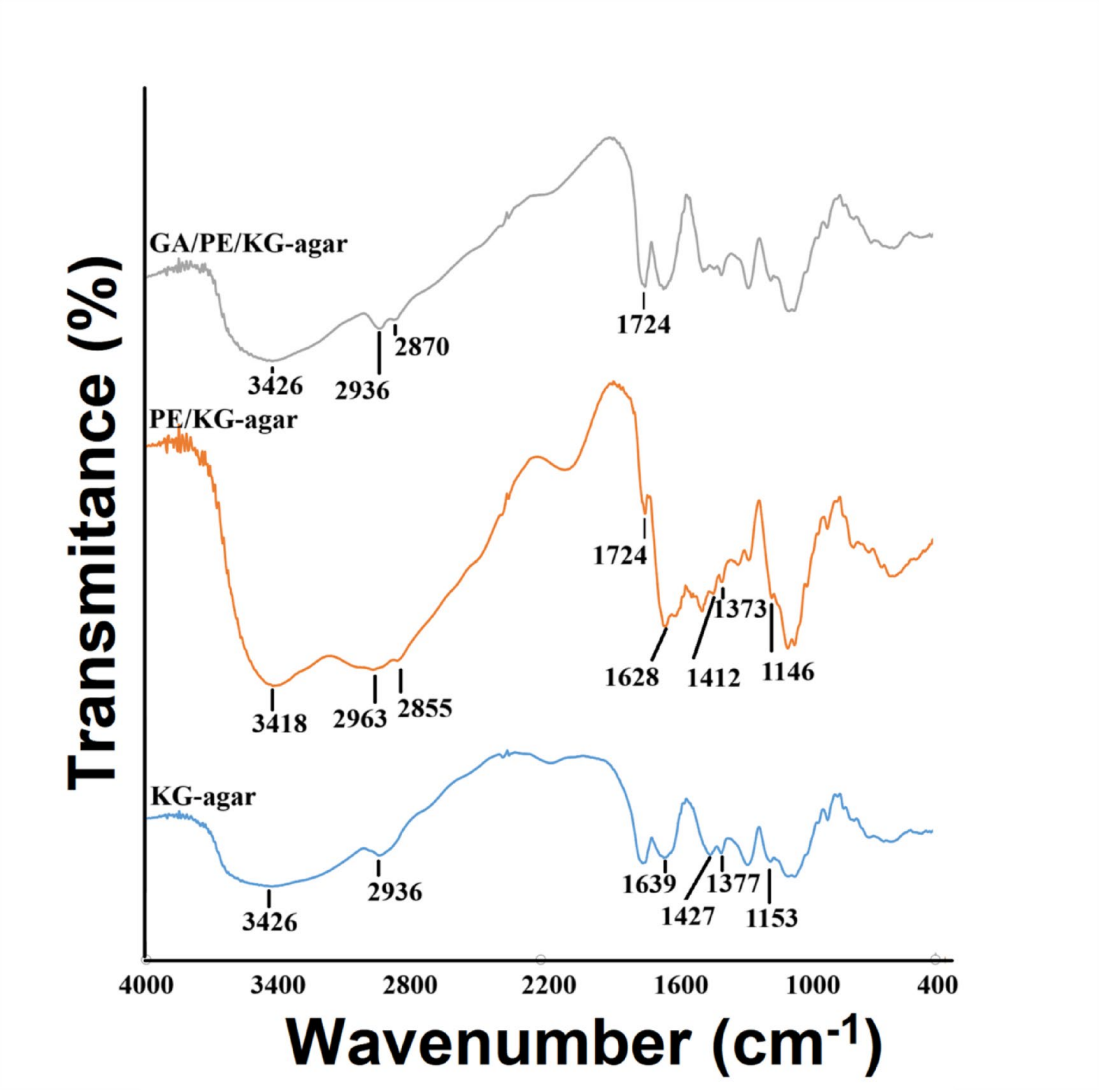
FTIR spectra of the KG-agar, optimal PE/KG-agar, and optimal GA/PE/KG-agar discs.

The broad 3426 cm^−1^ band in the KG-agar discs spectrum (Fig. 5) could be assigned to the stretching vibrations of the bounteous OH moieties^29^ of both KG and agar. On the other hand, the 2936 cm^−1^ band could be assigned to the stretching vibrations of the polysaccharides skeletal CH and CH_2_ moieties^29^. Noteworthy, KG comprises galacturonic and also glucuronic acid residues^7^. These acid residues were represented via their COO^−^ asymmetric stretching (1640–1610 cm^−1^)^30^ band at 1639 cm^−1^ and also via their COO^−^ symmetric stretching (1440–1360 cm^−1^)^31^ bands at 1427 and 1377 cm^−1^ (Fig. 5). Likewise, Dhua et al.^32^ previously assigned the 1427 cm^−1^ peak in KG spectrum to COO^−^ symmetric stretching. With respect to the characteristic agar peaks, prior FTIR analyses identified a peak at 1151 cm^−1^, which was assigned to the vibration of agar ester sulfate bond^33^. In Fig. 5, this peak was evident at 1153 cm^−1^.

Noteworthy, the FTIR spectrum of PE was previously presented, and it comprised bands at 2942 cm^−1^ and 2831 cm^−1^^34^. These bands could be assigned to the stretching vibrations of the CH_2_ moieties^29^ of PE. Moreover, the CH_2_ moieties bending vibrations were presented by the 1473 cm^−1^ band^34,35^. The previous PE spectrum also comprised bands at 3284 cm^−1^ and 1575 cm^−1^^34^, which could be assigned to the stretching and bending vibrations of the secondary amine N–H^35^, respectively. The 3284 cm^−1^ PE band^34^ merged with the broad KG-agar OH–stretching band (Fig. 5), resulting in a modification of the band area and its translocation to 3418 cm^−1.^ in the PE/KG-agar spectrum (Fig. 5). Moreover, the modifications observed in the OH-stretching band of the PE/KG-agar might infer that hydrogen-bonding was involved in PE binding to the KG-agar discs as it was previously reported that variations in OH-stretching bands could reflect variations in hydrogen-bonding^29^. Likewise, the variations observed in the OH-stretching bands of chia-gum films and calcium pectinate/whey-protein-isolate beads following their immersion in PE, suggested the role of hydrogen-bonding in PE binding to these bio-polymeric materials^8,9^. The PE binding to the KG-agar discs also involved ionic interactions amongst cationic PE and anionic KG-agar. This could be verified from the trans-location of KG COO^−^ asymmetric and symmetric stretching bands to 1628 cm^−1^ and 1412 &1373 cm^−1^, respectively (Fig. 5). Concurrently, the sulfate-related peak of agar at 1153 cm⁻^1^ shifted to 1146 cm⁻^1^, indicative of structural perturbation due to ionic association. Analogous shifts in chia gum COO^−^ bands after the ionic interaction with the cationic PE were also previously reported^9^. Noteworthy, the 2942 cm^−1^ and 2831 cm^−1^ bands, which were evidenced in the previous PE spectrum^34^, could have overlapped with KG-agar 2936 cm^−1^ band, resulting in the attainment of two bands at 2963 and 2855 cm^−1^ in the PE/KG-agar spectrum.

GA could be present in a linear or in a cyclic hemiacetal configuration. The former configuration comprises aldehyde residues whereas the latter comprises OH residues^16^. Evidence for the existence of both aldehyde and hydroxyl functionalities in GA could be derived from a previously recorded GA FTIR spectrum, which comprised a peak at 1722 cm^−1^, attributable to the carbonyl of aldehyde. This spectrum also comprised a wide band at ≈3530–3300 cm^−1^^36^, which could be assigned to the stretching vibrations of the OH moieties^29^ in GA. In the present study, the GA broad OH-stretching band overlapped with the broad OH-stretching band of the PE/KG-agar, resulting in a modification of the band area and its translocation to 3426 cm^−1^ (Fig. 5). Moreover, the GA aldehyde peak (1722 cm^−1^)^36^ overlapped with the 1724 cm^−1^ PE/KG-agar peak, resulting in significant intensification of the combined absorption feature.

### Chemical stability of the GA/PE/KG-agar immobilizers

Analyzing the citrate–phosphate buffer, in which the GA/PE/KG-agar discs were soaked for 19 h, revealed the absence of detectable GA, as optical density readings closely matched those of the blank. This indicated that GA did not leach from the chemically stable GA/PE/KG-agar discs. Such observed stability aligned with prior findings emphasizing the stability of GA-amino products. Specifically, it was previously established that proteins lysine residues, once modified by GA, did not regenerate even after being soaked for 24 h in 6 M HCl at 110 °C^16^.

### Effect of varying the loaded β-GL activity

Escalating the loaded β-GL activity from 5.99 to 29.79 Ug^−1^ was coupled with gradual escalations in both the iβ-GL activities and the immobilization yields (IYs) (Table 4). The escalations in the IYs indicated that more β-GL entities were conjugated to the GA/PE/KG-agar even after escalating the loaded β-GL activity by 4.97 fold. This implied that the GA/PE/KG-agar possessed a high density of reactive functional groups capable of accommodating greater β-GL loads without saturation. On the contrary, Kumar et al.^37^ reported consecutive drops in the IYs, provided by the grafted-silicon-dioxide-nanoparticles, after gradually escalating the loaded β-GL activity. These previously recorded IY drops could indicate the limited content of the available binding sites and their subsequent depletion at higher β-GL concentrations. Noteworthy, prior research^8^ utilizing GA/PE/(calcium pectinate/whey-protein-isolate) beads reported an IY of 35.50% at a loading of 29.57 Ug^−1^, a value closely aligned with the results obtained in the present study (Table 4).

The immobilization efficiency (IE), calculated as the percentage of active β-GL relative to the total conjugated β-GL, exhibited progressive decline from 68.73% to 35.94% upon escalating the loaded β-GL activity (Table 4). This indicated that higher loading levels resulted in a greater proportion of inactivated iβ-GL entities. This could be a consequence of the crowding amongst the escalated amounts of iβ-GL. Such crowding would facilitate the incidence of protein–protein reactions^38^, which could be inactivating. Furthermore, crowding might obstruct the enzyme capability to undergo its essential configuration modifications, and this would also inactivate a portion^38^ of the iβ-GL. Similar reductions in β-GL IEs with increased loading were previously observed when using the GA/PE/chia gum and the GA/PE/(calcium pectinate/whey-protein-isolate) as immobilizers^8,9^. Importantly, the lowest IE value obtained in this study was higher than the 22% β-GL IE, which was recorded by the optimal genipin grafted (alginate-gelatin) immobilizers^39^.

### pH profiles inspection

**Fig. 6:**
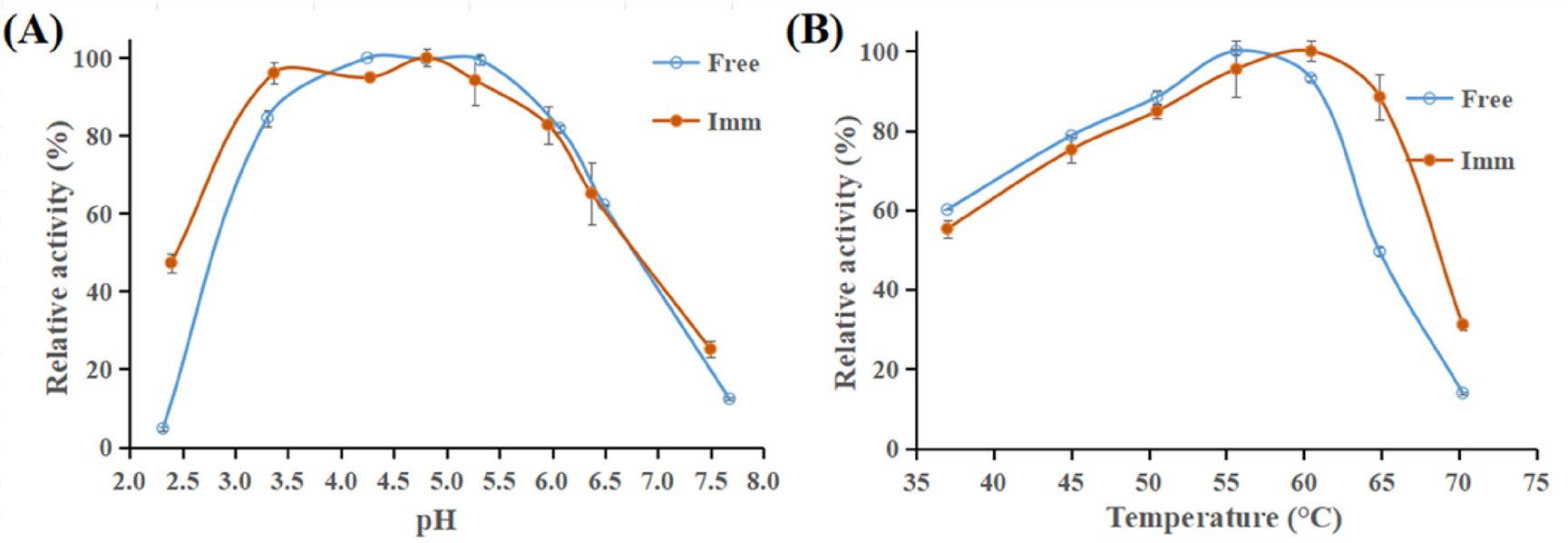
(**A**) pH activity profiles and (**B**) temperature activity profiles of the GA/PE/KG-agar iβ-GL and its free homologue (Mean ± S.E.).

The free β-GL uppermost activities were provided within 4.3–5.3 pH range (Fig. 6A). This was in alignment with earlier reports which placed *A. oryzae* β-GL optimal pH amongst the 4.5–5.5 pHs^40–42^. With respect to the GA/PE/KG-agar iβ-GL, it provided 96.1%, 95.0%, 100.0%, and 94.25% activities at pHs 3.4, 4.3, 4.8, and 5.3, respectively. Thus, the 3.4–5.3 pH range could be regarded optimal for the GA/PE/KG-agar iβ-GL, and this reflected an acidic shifting. Likewise, entrapping the *A. oryzae* β-GL within variable silica composites, shifted its optimal pH from 5 to 4.4^43^. Furthermore, β-GL covalent conjugation to the GA/copper-gelled-chitosan and GA/PE/chia gum shifted its optimal pH from 4.4–5.5 to 3.6–4.4 and from 4.8–5.2 to 3.3–4.9, respectively. Such acidic shifting was previously referred to the cationic nature of the immobilizers^9,44^, a factor likely applicable to the GA/PE/KG-agar immobilizers as well, given the positively charged functional entities introduced by the PE coating. Ionic interactions could elicit between the immobilizer cationic entities and the anionic hydroxyl species in solution. As a result, the hydroxyl species might accumulate near the immobilizer surface, increasing their local concentration. Such redistribution of hydroxyl ions was hypothesized in earlier literature to induce a lowering of the enzymes optimal pH^9,44^.

### Temperature profiles inspection

The free β-GL uppermost activity was provided at 56 °C (Fig. 6B). This finding aligned with prior observations by Gao et al.^40^, who reported that 55 °C was the optimal for the *A. oryzae* β-GL. With regards to the GA/PE/KG-agar iβ-GL, its optimal temperature range was widened to 56–60 °C. The iβ-GL also provided 88.47% and 31.09% activities at 65 °C and 70 °C, respectively. These values were more escalated than the 49.53% and 13.88% activities provided by the free β-GL under analogous conditions. These results indicated that the GA/PE/KG-agar iβ-GL was more thermo-tolerant. The multi-point covalent links created amidst the iβ-GL and the GA in the GA/PE/KG-agar rigidified the enzyme configuration, making it more resistive against distorting factors^11^, such as raised temperatures. Consistent with these findings, previous investigations reported elevated optimal temperatures for β-GL following its entrapment within variable silica composites^43^, as well as following its covalent conjugation to the genipin grafted (alginate-gelatin) immobilizers^39^.

### Thermal stability

**Fig. 7:**
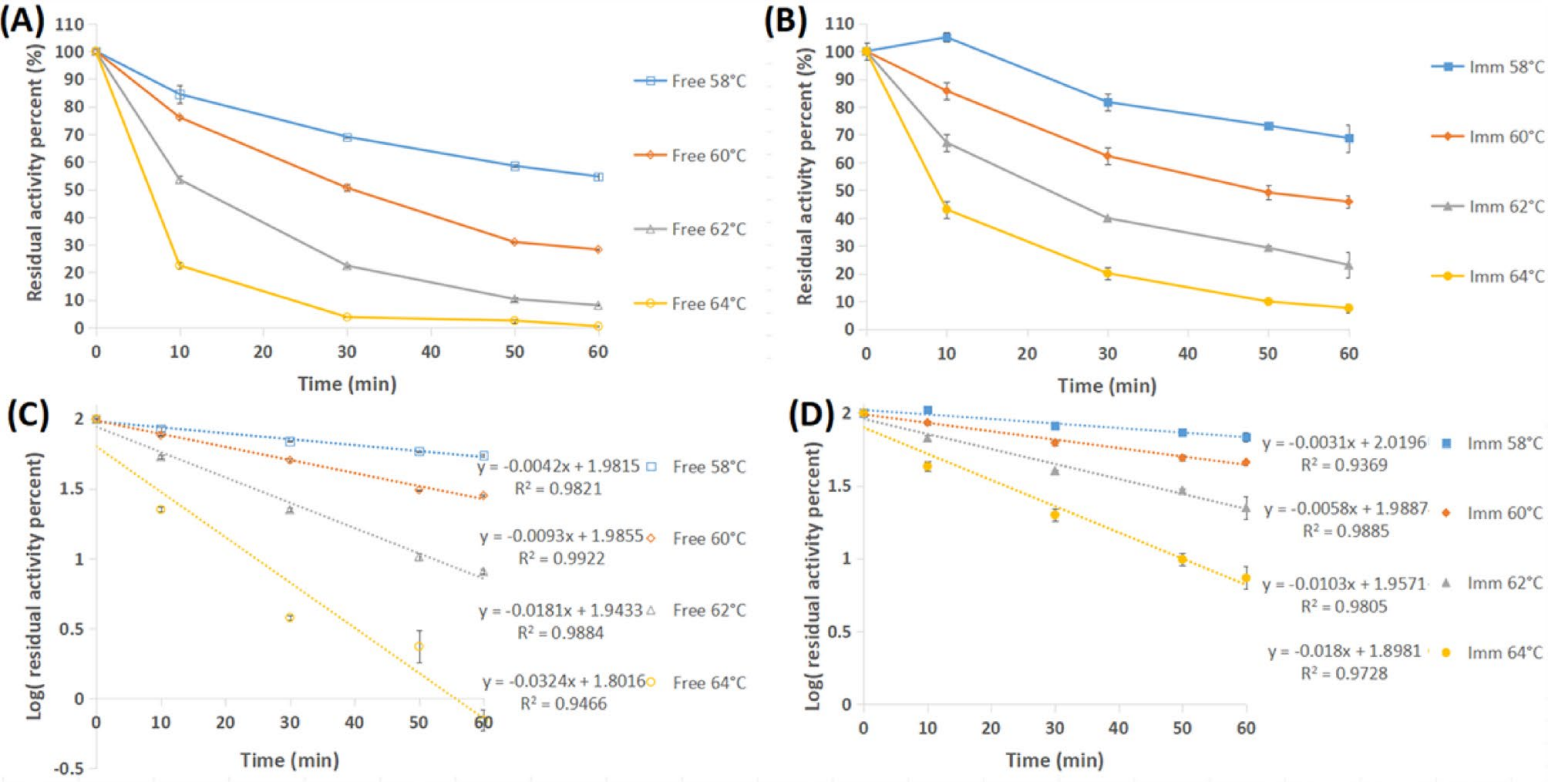
Percents of (**A**) the free and (**B**) the GA/PE/KG-agar iβ-GLs residual activities, which were provided following their thermal incubations. Plots to estimate k_d_ (-slope) for the (**C**) the free and (**D**) the GA/PE/KG-agar iβ-GLs. (Mean ± S.E.).

The GA/PE/KG-agar iβ-GL exhibited finer thermal stability than its free homologue. The GA/PE/KG-agar iβ-GL conserved 68.75%, 45.86%, 23.09% and 7.59% activities after 1 h of incubation at 58, 60, 62, and 64 °C, respectively. On the other hand, only 54.71%, 28.25%, 8.08%, and 0.47% activities were conserved, respectively, by the free β-GL after similar incubations (Fig. 7A and B). Likewise, the β-GLs, which were covalently conjugated to the GA/diaminopolyethylene-glycol-polydopamine processed magnetic nano-particles and to the GA/PE/chia gum immobilizers, conserved larger activity percents than did their free homologues after thermal incubations^9,41^. The finer thermal stability of the GA/PE/KG-agar iβ-GL could be regarded to the multi-point covalent links betwixt the GA residues of the GA/PE/KG-agar and the iβ-GL as these links would rigidify the enzyme configuration, making it more resistive against distorting factors^11^, such as raised temperatures.

The finer GA/PE/KG-agar iβ-GL thermal stability could also be confirmed from Table 5. The k_d_-values recorded for the GA/PE/KG-agar iβ-GL were lesser than those recorded for its free homologue at all given temperatures, and this implied that GA/PE/KG-agar iβ-GL thermal denaturation occurred at a slower pace. Moreover, the GA/PE/KG-agar iβ-GL t_1/2_ and D-values were 1.37, 1.62, 1.76, and 1.80 folds more escalated than those offered by the free β-GL at 58, 60, 62, and 64 °C, respectively. Such escalated t_1/2_ and D-values implied that more extended thermal incubations would be needed for the GA/PE/KG-agar iβ-GL activity to drop by 50% and 90%, respectively. Noteworthy, the finer thermal stability conferred to β-GL, following its covalent conjugation to the GA/egg-white-protein processed gellan gum, was also confirmed from its lesser k_d_-values, and its more escalated t_1/2_ & D-values^45^. Furthermore, prior studies demonstrated a 1.30-fold enhancement in both t_1/2_ and D-value of β-GL at 60 °C following its covalent conjugation to the GA/amino-processed-magnetic-mesoporous silica^40^.

**Table 5:** Thermodynamic parameters of the GA/PE/KG-agar iβ-GL and its free homologue determined from thermal incubation studies.

Temp (°C)	K_d_ (min^1^)		t_1/2_ (min)		D-value (min)		ΔH° (kJ mol^−1^)		ΔG° (kJ mol^−1^)		ΔS° (J mol^−1^ K^−1^)	
	Free	Imm	Free	Imm	Free	Imm	Free	Imm	Free	Imm	Free	Imm
58	0.0042	0.0031	163.17	223.16	542.03	741.33	311.13	268.70	107.70	108.56	614.30	483.58
60	0.0093	0.0058	74.23	120.04	246.58	398.76	311.11	268.68	106.18	107.52	615.11	483.77
62	0.0181	0.0103	38.24	67.39	127.04	223.85	311.09	268.67	104.99	106.57	614.95	483.65
64	0.0324	0.0180	21.38	38.51	71.01	127.93	311.08	268.65	104.00	105.65	614.18	483.45

**Fig. 8:**
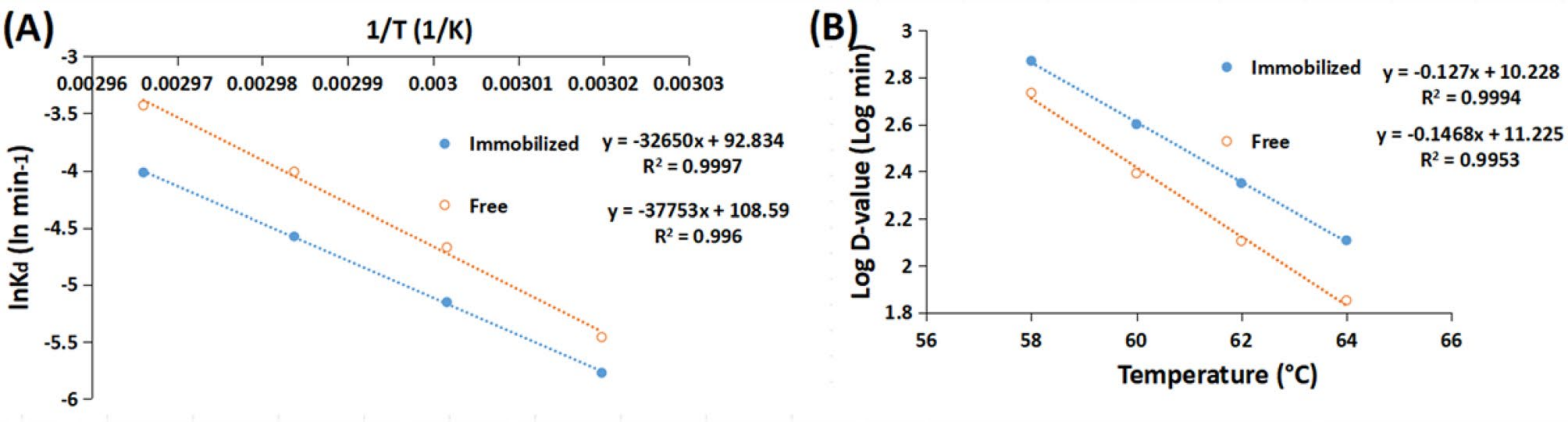
(**A**) Arrhenius plots adopted to reckon E_d_ and (**B**) plots to estimate z-values (-1/slope) for the β-GL specimens. (Mean ± S.E.).

The free and the GA/PE/KG-agar iβ-GLs E_d_-values were calculated from the slopes in Fig. 8A, and they amounted to 313.88 and 271.4 kJ mol^−1^, respectively. This indicated a reduction in β-GL E_d_-value following its conjugation to the GA/PE/KG-agar. A comparable reduction in β-GL E_d_-value, from 324.36 to 279.58 kJ mol^−1^, was previously recorded after β-GL covalent conjugation to the GA/PE/chia gum^9^. Moreover, 1.63 and 3.17 fold drops were recorded in the E_d_-values of carboxy-methyl-cellulase and alkaline protease after their immobilization^25,46^. Such E_d-_value drops were regarded to the lesser temperature sensitiveness of the more thermally-stable immobilized enzymes^9,25,46^.

The lesser temperature sensitiveness of the GA/PE/KG-agar iβ-GL was further reflected in an escalated z-value of 7.87 °C, compared to a z-value of 6.81 °C for the free β-GL This indicated that a 7.87 °C temperature escalation would be required to reduce the GA/PE/KG-agar iβ-GL D-values by 90% whereas a temperature escalation of only 6.81 °C would be required to induce the selfsame effect in case of the free β-GL. Noteworthy, z-value escalations were frequently reported following enzymes immobilization^9,25,46^.

The finer thermal stability of the GA/PE/KG-agar iβ-GL was further substantiated by its ΔS and ΔG (Table 5). ΔS reflects systems randomness, and it is stated that the enzymes, which are more stable, provide lesser ΔS^47^. In the present study, the GA/PE/KG-agar iβ-GL exhibited consistently lesser ΔS values across all tested temperatures compared to the free β-GL, thereby confirming its improved thermal stability. This result was consistent with earlier reports demonstrating that the more thermally stable immobilized forms of carboxy-methyl-cellulase, alkaline protease, and β-GL exhibited reduced ΔS values relative to their free homologues^9,25,46^.

With respect to ΔG, as it combines both enthalpic and entropic terms, it serves as a more reliant parameter for assessing enzymes stability. Enzymes with improved thermal stability and resistance to denaturation typically display more escalated ΔG values^47^. In this context, the GA/PE/KG-agar iβ-GL exhibited consistently more escalated ΔG values compared to free β-GL at all tested temperatures. Similarly, a prior study reported that β-GL, which was covalently conjugated to to GA/egg-white-protein processed gellan gum, also provided more escalated ΔG than did its free homologue^45^.

### Influence of heavy-metals on the stability of the free and the GA/PE/KG-agar iβ-GLs

Environmental pollution with heavy-metals could lead to milk contamination because livestock could be exposed to these heavy-metals through inhalation or ingestion of polluted water or vegetation^48,49^. Evidence from various studies confirmed the presence of lead and mercury in milk specimens collected in China, Italy, Egypt, and Banglades^48^. Aluminium was also detected in milk specimens from Turkey, China, and France^50^. Moreover, analyzing 195 milk specimens from different locations in the Kvemo Kartli region, Georgia, revealed that all specimens contained chromium, iron, and lead^49^. The milk contaminating heavy-metals might transfer into whey during cheese manufacturing. Thus, the whey, which is the principal substrate for the *A. oryzae* β-GL^17^, could be contaminated with heavy-metals. Moreover, if the whey was utilized for GOS production, it would be concentrated in order to raise its lactose content^51^, and this would further increment the concentration of the contaminating heavy-metals. It should be noted that if the heavy-metal levels surpass the established safety thresholds, remedial treatment will be necessary prior to human consumption. However, even after such treatments, residual metal-ions may persist, underscoring the need for a β-GL exhibiting enhanced tolerance to heavy-metals. Therefore, the influence of these heavy-metal ions on the stability the free and the GA/PE/KG-agar iβ-GLs was inspected.

**Fig. 9:**
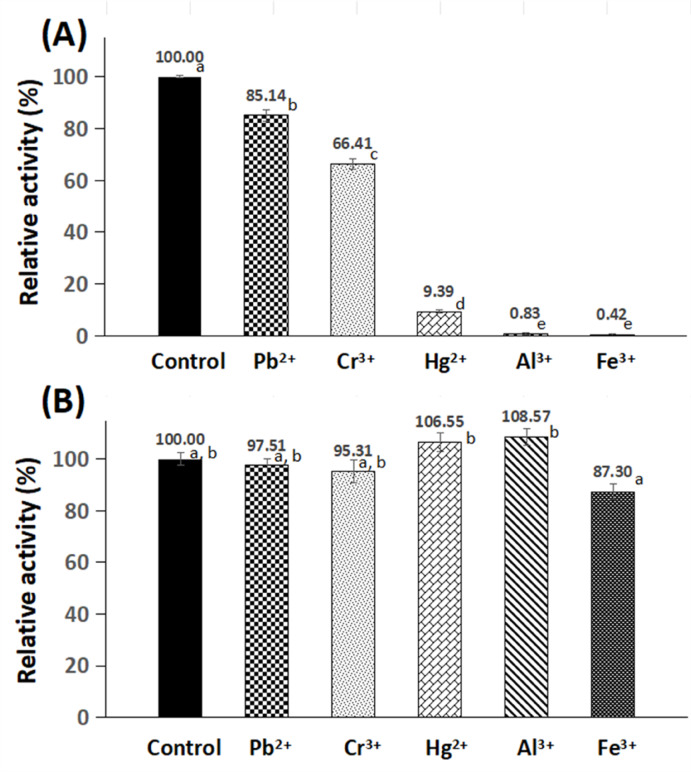
Stability of (**A**) the free and (**B**) the GA/PE/KG-agar iβ-GLs amongst varied metal ions. (Mean ± S.E.; variable small case letters indicated statistically significant results, *p*-value < 0.05).

The free β-GL was significantly inhibited by all listed heavy-metal ions, with mercury, aluminium, and iron exhibiting particularly pronounced inhibitory effects, resulting in residual activities of 9.39%, 0.83%, and 0.42%, respectively (Fig. 9). These findings aligned with prior studies documenting the potent inhibition of *A. oryzae* β-GL by mercury, aluminium, and iron^9^. In contrast, the activity of the GA/PE/KG-agar iβ-GL wasn’t significantly (*p*-value < 0.05) altered by any of the listed heavy-metal ions (Fig. 9). Such robust stability might be ascribed to the thick PE coating which was formulated around the KG-agar discs at the optimal 6.1% PE concentration. The enzyme would penetrate into this PE coating^15^, and this would partition it away from the heavy-metal ions in the bulk solution. Moreover, PE becomes more cationic as its pH drops^52^. Thus, at pH 4.8, which was adopted during β-GL activity inspection, the cationic PE would electrostatically repel the cationic heavy-metal ions, partitioning them away from the iβ-GL moieties, and this would promote the GA/PE/KG-agar iβ-GL stability in presence of these heavy-metal ions. Noteworthy, the β-GL, which was covalently conjugated to the GA/PE/chia gum, also provided finer stability versus chromium, iron, and aluminium than did its free homologue^9^.

### Solvent stability of the free and the GA/PE/KG-agar iβ-GLs

**Fig. 10:**
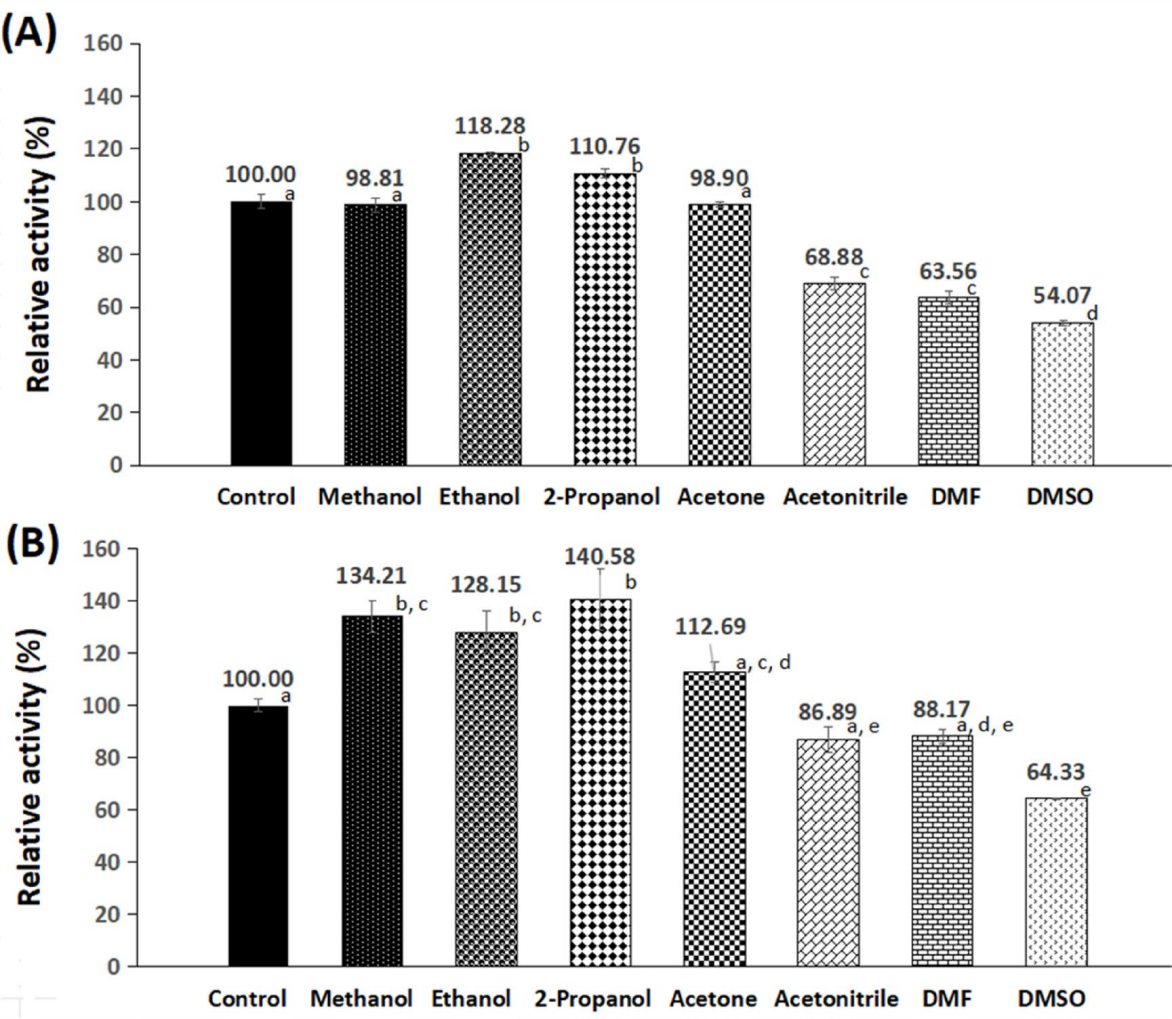
Stability of (**A**) the free and (**B**) the GA/PE/KG-agar iβ-GLs amongst varied solvents. (Mean ± S.E.; variable small case letters indicated statistically significant results, *p*-value < 0.05).

The free β-GL activity was significantly (*p*-value > 0.05) inhibited by acetonitrile, DMF, and DMSO, retaining only 68.88%, 63.56%, and 54.07% activities, respectively (Fig. 10). This aligned with prior studies documenting the inhibitory effects of acetonitrile, DMF, and DMSO on the *A. oryzae* β-GL^9,44^. In contrast, GA/PE/KG-agar iβ-GL demonstrated significant inhibition solely in the presence of DMSO (64.33%), while its exposure to acetonitrile and DMF resulted in insignificant activity drops (Fig. 10). This indicated the finer solvent stability of the GA/PE/KG-agar iβ-GL, which could be referred to the thick PE coating that encircled the KG-agar discs. The hydrophilic PE was previously argued to promote enzymes stability via partitioning detrimental moieties, such as organic solvents, away from such enzymes^15^. Furthermore, immobilizers could serve as water-storage, and this would prevent organic solvents from strapping the enzymes vital water layer^11^. Noteworthy, β-GL immobilization has previously promoted its solvent stability versus altered solvents, such as acetonitrile, DMSO^53^, and DMF^9^.

It was also disclosed that neither the free nor the GA/PE/KG-agar iβ-GLs were significantly inhibited by methanol, ethanol, and 2-propanol. The free β-GL provided 98.81%, 118.28%, and 110.76% activities, respectively, and more escalated activity percents of 134.21%, 128.15%, and 140.58%, respectively, were provided by the GA/PE/KG-agar iβ-GL. Dragosits et al.^54^ previously highlighted that methanol, ethanol, and 2-propanol influences on the *A. oryzae* β-GL activities were concentration reliant. Initially, both methanol and ethanol considerably promoted β-GL activity. Nonetheless, when their concentrations were escalated beyond certain values, the β-GL activity dropped. The situation was analogous in case of 2-propanol, but only a slight activity escalation was recorded at the initial inspected 2-propanol concentration, afterwards, activity drops were recorded with the more escalated 2-propanol concentrations^54^. In the present study, the escalated activity percents provided by the free β-GL implied that the adopted solvent concentrations were below the inhibitory threshold. Furthermore, in the case of the GA/PE/KG-agar iβ-GL, effective solvent concentrations at the enzyme micro-environment were likely reduced due to the presence of the thick PE coating that encircled the KG-agar discs. The enzyme would penetrate into this PE coating^15^, and this would partition it away from the solvents in the bulk solution, and accordingly, the effective solvent concentrations would be lowered. These lowered methanol, ethanol, and 2-propanol concentrations significantly promoted the GA/PE/KG-agar iβ-GL activity. Likewise, the activity of the GA/copper-gelled-chitosan iβ-GL was significantly promoted by methanol, ethanol, and 2-propanol^44^.

### GA/PE/KG-agar iβ-GL storage stability

**Fig. 11:**
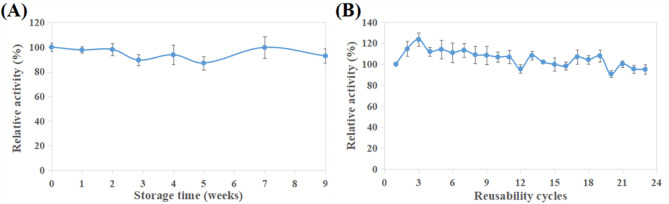
(**A**) storage stability and (**B**) reusability of the GA/PE/KG-agar iβ-GL. (Mean ± S.E.).

The GA/PE/KG-agar iβ-GL provided 93.77% and 92.86% activities after 4 and 9 weeks storage periods, respectively (Fig. 11A). Such storage stability was finer than that provided by the β-GL, which were covalently conjugated to the GA/diaminopolyethylene-glycol-polydopamine processed magnetic nano-particles, as it provided only 63.70% following 8 weeks storage period^41^. In comparison, the β-GL, which was adsorbed by the nano-silver grafted reduced-graphene-oxide, provided 85% activity following a 60 days storage period^42^. The fine storage stability of the GA/PE/KG-agar iβ-GL could be regarded to the multi-point covalent links betwixt the GA residues of the GA/PE/KG-agar and the iβ-GL as these links rigidified the enzyme configuration^11^, thereby, prohibiting significant alterations in the enzyme active site^42^.

### Reusability of the GA/PE/KG-agar iβ-GL

Upon reusing the GA/PE/KG-agar iβ-GL, its activity went up to 114.63% and 123.66% during the first and second cycles, respectively (Fig. 11B). A similar enhancement was observed in the activity of the β-GL, which was covalently conjugated to GA/egg-white-protein processed gellan gum, during its initial two reusability cycles. It was argued that the immobilized enzyme had to be incubated with its substrate for some time before achieving its uttermost activity^45^. Upon further reusing the GA/PE/KG-agar iβ-GL, its activity dropped (Fig. 11B). Nonetheless, 111.04%, 106.83%, 99.91% and 95.11% activities were presented during the 6th, 10th, 15th, and 23rd cycles. On the other hand, it was previously stated that the gluconic acid grafted fullerenes iβ-GL presented only 89% activity during its 6th cycle^55^. Furthermore, the β-GLs, which were covalently conjugated to the GA/diaminopolyethylene-glycol-polydopamine processed magnetic nano-particles and the GA/amino-processed-magnetic-mesoporous silica, presented 81% and 86.4% activities, respectively, throughout their 10th cycles^40,41^. The fine operational stability of the GA/PE/KG-agar iβ-GL could be regarded to the multi-point covalent links betwixt the GA residues of the GA/PE/KG-agar and the iβ-GL as these links rigidified the enzyme configuration, making it more resistant versus distorting factors^11^, such as the recurrent interactions with the substrate as these interactions might be distorting to the active site^9^.

### Degrading whey permeate lactose via the free and the GA/PE/KG-agar iβ-GLs

**Fig. 12:**
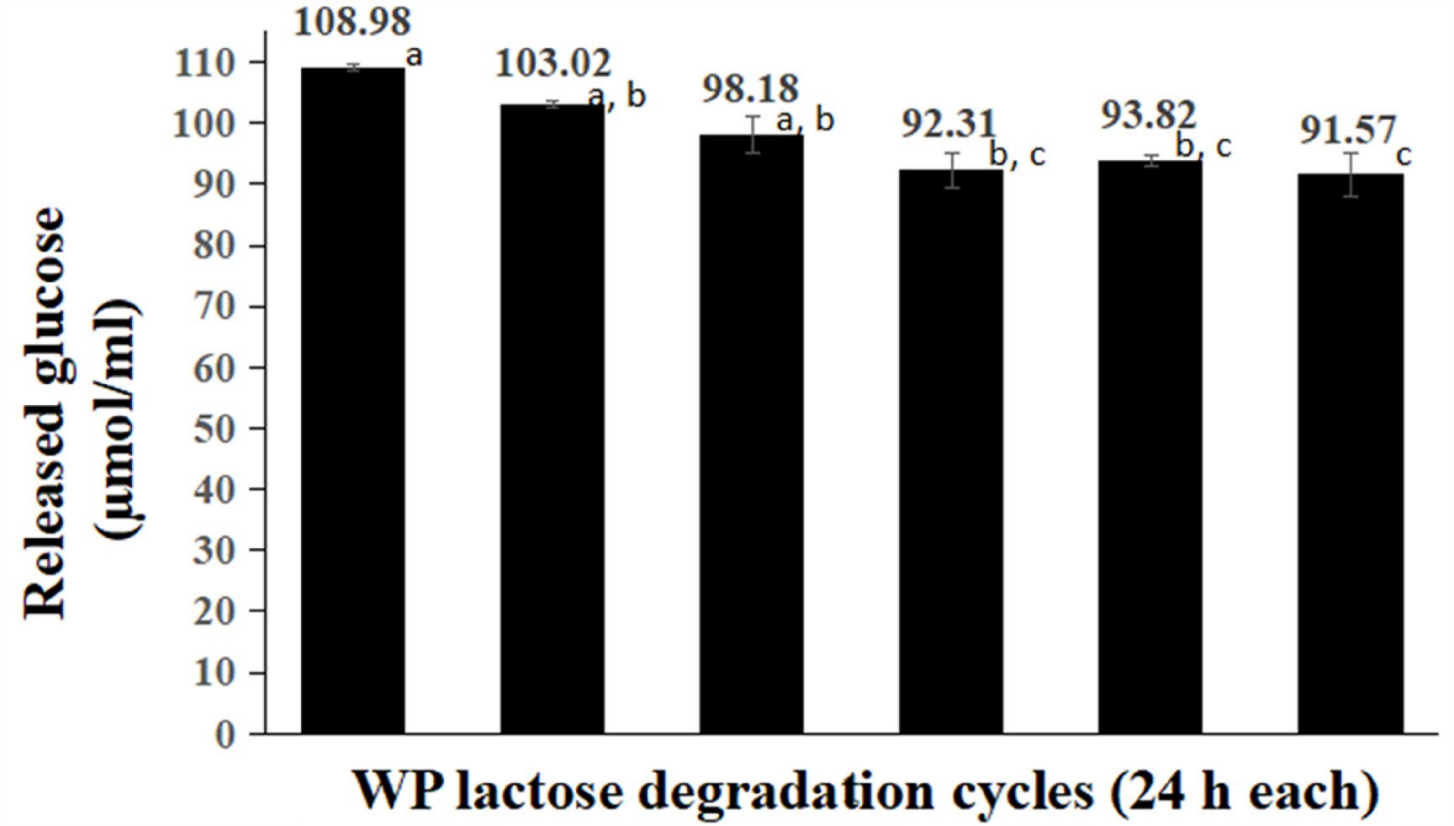
Glucose released after processing WP with the GA/PE/KG-agar iβ-GL for 6 successive 24 h lactose degradation cycles. (Mean ± S.E.; variable small case letters indicated statistically significant results, *p*-value < 0.05).

Figure 12 disclosed that 108.98 µmol/ml glucose was detected after treating the WP with the GA/PE/KG-agar iβ-GL for 24 h. This corresponded to 79.42% lactose degradation proficiency (LDP) as the WP originally contained 137.23 µmol/ml lactose. In contrast, the *A. oryzae* β-GLs, which were coupled to the GA/PE/chia gum immobilizers and the magnetic nano-cellulose, achieved only 57.54% and > 50% LDP, following 24 h incubation with WP and whey, respectively^9,56^. It was previously argued that the continuous accruement of glucose and galactose, which are β-GL noncompetitive and competitive inhibitors, respectively, would prevent full lactose breakdown. Additionally, whey minerals may inhibit β-GL activity, which would also prevent full lactose breakdown^57^.

When the free β-GL was used to degrade WP lactose, only 67.50 ± 0.58 µmol/ml glucose was detected, which corresponded to 49.49% LDP. This performance was inferior to that of the GA/PE/KG-agar iβ-GL, thereby confirming the enhanced efficacy of the iβ-GL. The superior performance of the iβ-GL might be partially ascribed to the escalated thermal stability which helped preserve its activity during the extended incubation at 45 °C. It should also be noted that the reuse of the free enzyme was unfeasible due to inability to recover it post-reaction. Conversely, the GA/PE/KG-agar iβ-GL was readily recovered, and accordingly, it was utilized for successive WP degradation cycles.

During the successive GA/PE/KG-agar iβ-GL mediated WP degradation cycles, glucose yields gradually declined. Nonetheless, 91.57 µmol/ml glucose was detected during the 6th degradation cycle, amounting to 84.02% of the yield observed in the first degradation cycle (Fig. 12). By comparison, systems employing the β-GLs covalently conjugated to the GA/copper-gelled-chitosan and the GA/PE/(calcium pectinate/whey-protein-isolate) immobilizers provided only 50.53% and 73.58% of their initial glucose yields by their fifth and sixth whey lactose degradation cycles, respectively.

## Conclusions

KG derived covalent immobilizers were prepared herein for the first time. Initially, a straightforward method involving the integration of agar was employed to formulate KG as a handle-able hydrogel. This KG-agar hydrogel was subsequently processed with PE and GA in order to obtain the GA/PE/KG-agar covalent immobilizers, which effectively immobilized β-GL with IEs reaching 68.73%. The iβ-GL exhibited significantly enhanced thermal, solvent, and heavy-metals stabilities as compared to its free homologue. Moreover, the GA/PE/KG-agar iβ-GL provided robust storage and operational stabilities with 92.86% activity provided after 9 weeks storage and 95.11% activity provided during its 23rd reusability cycle. The GA/PE/KG-agar iβ-GL was also successfully applied in a key industrial process, which was the degradation of WP lactose, and this confirmed its practical utility in biotechnological applications.

It should be noted that the GA/PE/KG-agar immobilizers could only be fabricated in disc or film configurations due to the intrinsic gelling properties of agar, which undergoes a coil-to-helix structural transition upon cooling, resulting in solidification^13^. However, alternative physical forms might be necessary depending on the intended application. For example, when immobilized biocatalysts are to be employed in packed-bed reactors, a bead-like morphology is essential for effective column packing, Carrageenan (Car) is a mechanically stable, anionic hydrogel^26^, which solidifies on cooling after a coil-to-helix configuration transformation^58^, and also forms beads via potassium mediated gelation^26^. Consequently, Car-based hydrogels offer versatility in morphology, allowing the production of discs, films, and also beads. Thus, future work on KG based covalent immobilizers might involve the integration of Car with KG in order to obtain beads and discs, which can be subsequently activated into covalent immobilizers.

## Data Availability

The author states that the data needed to reproduce the findings of this research are provided within the article.
